# Melange

**Published:** 1885-12

**Authors:** 


### MELANGE.

STONES AND GLASS HOUSES—Our witty and somewhat savage contemporary of the Louisville *Medical Herald* accuses the editor of the New York *Medical Record* of having "ramblissment of the brain," reads him a lesson in ethics, and exhorts him to "confess his wrongs and come back to the fold," etc. The *Record* doesn't "tumble" to the "ramblissment," and declines to "confess his wrongs;" but "comes back" at his assailant neatly, by asking if advocating the old code on one page and advertising a *homœopathic journal* on the next, is just his idea of "ethics." Pretty good.

---

HYMENEAL.—Dr. A. H. Caldwell, of The Grove, Texas, was married on the 28th of October to Miss Imogin Smith, daughter of A. F. Smith, Esq., of same place. We extend to the Doctor our warmest congratulations, and bespeak for him and his bride all the happiness and prosperity they could desire.

